# Diagnosis of Dilated Cardiomyopathy: Patient Reaction and Adaptation—Case Study and Review of the Literature

**DOI:** 10.1155/2016/1756510

**Published:** 2016-10-16

**Authors:** Solomis Solomou, Maria Stavrou, Justin Marley

**Affiliations:** ^1^Barnet, Enfield and Haringey Mental Health NHS Trust, London N15 3TH, UK; ^2^Royal London Hospital, Barts Health NHS Trust, Whitechapel Road, London E1 1BB, UK; ^3^The Kingswood Centre, North Essex Partnership University NHS Foundation Trust, Turner Road, Colchester CO4 5JY, UK

## Abstract

*Objective*. Heart failure remains a major cause of morbidity and mortality. Given that heart failure generally has a chronic course, it is important to appreciate the impact it can have on the quality of life of patients and also their partners or family carers.* Method*. Questionnaires were given to a patient newly diagnosed with dilated cardiomyopathy, during his hospital admission, as well as after discharge. The responses are summarised and explored in the discussion section, where we used review of the literature to discuss the implications of a new diagnosis of heart failure.* Results.* The patient's responses to the questionnaires suggest certain anxieties that are part of his adaptation to the diagnosis of heart failure.* Conclusion.* Depression is a common comorbid condition in patients with heart failure. Various tools can be used to screen for depression in patients with heart failure. Both pharmacological and nonpharmacological options are available. Rapid evaluation of ongoing problems and active participation by a psychiatrist can ensure that the patient receives the best possible clinical care.

## 1. Case Presentation

A 54-year old male self-presented to the emergency department with a 4-day history of awareness of irregularities of his heart rhythm. This was not accompanied by any chest pain but he revealed a one-month history of shortness of breath, tiredness, and leg swelling. He also reported general malaise and decreased libido. He had recently stopped smoking when the symptoms first started and used to smoke 15 cigarettes a day prior to that for 25 years. He had no prior medical or surgical history other than mild asthma for which he had not been hospitalized previously.

On admission, he was found to have a narrow complex tachycardia, which reverted to normal sinus rhythm with intravenous verapamil. Initial investigations showed Troponin I of 0.22 (normal < 0.04), a BNP of 899 (normal < 100), and raised D-dimers of 664. A CT pulmonary angiogram excluded a pulmonary embolus. A transthoracic echocardiogram showed mildly dilated left and right ventricles with global hypokinesia and severe biventricular systolic dysfunction. The left ventricular ejection fraction (LVEF) was calculated, by Simpson's method, to be 15% (normal > 55%). The echocardiogram also showed there was no left ventricular hypertrophy or valve disease of any significance. A full cardiomyopathy biomarker screen was negative for any secondary causes. Coronary angiography revealed only mild atheroma in the proximal left anterior descending and mid-right coronary arteries. The patient had no further rhythm disturbances on CCU and lost 6.2 kg in weight during his 5-day stay as an inpatient.

## 2. Patient's Reflections during Admission

On transfer from the emergency department to the coronary care unit, the patient said he did not feel anxious because he was in the right place. The diagnosis of dilated cardiomyopathy and biventricular congestive heart failure was given to the patient: “It was those last two words that got my attention. When I was told I was suffering from ‘heart failure' I was shocked. The heart is one muscle that can't regenerate itself.” He tried to find leaflets with simple information on heart failure in the ward, but found none. However, “All my questions were answered. The diagnosis was given to me in a clear and straightforward way.”

On direct questioning of his appreciation about the management of his condition, he replied: “The first thing to do, the consultant said, is to see how I do on an ACE inhibitor and a beta blocker, along with some other medicines for heart failure. And if those don't work well enough, there are other things to try such as implanted pacemakers to help the heart work better. The consultant mentioned the possibility of a heart transplant as the last resort; in other words my heart might as well be thrown away…”

During the patient's stay, the “Heart Improvement Team” specialist nurse was assigned to evaluate, inform, and educate him on his condition and emphasize medication adherence, mild salt and fluid restriction, and recognition of signs and symptoms that would indicate progression of the disease. These discussions enabled the patient to become “a strong partner in his treatment plan.” “I feel safe and I have the exact dates of the follow-up appointments with the consultant and the specialist nurse.”

The patient had to share his diagnosis with his two teenage children. For him, this was the “most difficult part of the journey,” not being sure of “how much to tell them.” He decided to be truthful and open, tailoring his approach to each child. The diagnosis proved to have long-term effects on his lifestyle choices, such as deciding to work part-time and making the decision to move in with his partner, plan their wedding, and “value each moment.”

## 3. Patient's Reflections One Month after Discharge

The patient explained that, during a general discussion with the GP about his diagnosis, the long-term prospects, life expectancy, and longevity were explained to him following discharge from hospital. He decided that his preference would be to control his condition with drugs. He was given advice regarding smoking, which he found very difficult to stop. After getting his diagnosis, the patient felt uncertain and alarmed after being told: “Your heart is not functioning properly.” He felt that the discussion on possible treatments was shocking; this included the percentage likelihood that he would need invasive procedures and the possibility of heart transplant and pacemaker, as well as continuing treatments, possible research trials, and gene therapy. The information that had the biggest impact on him was the possibility of a heart transplant. There was a gap between leaving the hospital and seeing the specialist Heart Improvement Team. The fact of not knowing and not having further contact and feedback made him feel anxious.

Apart from being given a questionnaire by a junior doctor on the ward, the discussion with the consultant cardiologist was the only discussion the patient had in the hospital about the impact of his diagnosis. It was not mentioned by any other member of staff. He felt it would have been helpful if this had not been the case. His partner was present when the diagnosis was disclosed. The patient thought it was helpful to have her there but he was concerned about how she would react to the information rather than focusing on his own feelings. He felt it would have been better if he had been given the diagnosis without his partner or anyone else present initially and then went over it again with her present, preferably with someone senior.

When asked how has this life event impacted his life, the patient explained that he had to bring forward some vital decisions in his life that he had been postponing for years, such as marriage and moving house. In terms of changes to his lifestyle, he explained he had a problem of lack of sexual interest and loss of libido. He felt worried and concerned about it, as he had never had a problem in terms of initiation and desire before. There were both positive and negative changes in terms of the way he perceived his image. He felt he looked better as he had lost weight since being on the diuretics. His negative perceptions related to the fact that he now has to spend his whole life with this condition.

When questioned whether the patient would consider counselling, he explained that, depending on the outcome of his treatment, it might be useful for him and his wife to have counselling in the future. He felt that the future seemed uncertain because of his lack of knowledge about how the condition is going to impact his longevity and felt uncertainty regarding the prospect of living after the age of 60 years.

## 4. Discussion

Heart failure remains a major cause of morbidity and mortality [1] with increasing prevalence due to the wider availability of biomarkers for diagnosis and easier access to echocardiography [2]. Given that heart failure generally has a chronic course, with the exception of some cardiomyopathies and valvular related disease, it is important to appreciate the impact it can have on the quality of life of patients and also their partners or family carers.

The patient's responses to the questionnaires suggest certain anxieties that are part of his adaptation to the diagnosis of heart failure. These anxieties relate to the fact that he had limited knowledge of what heart failure is before being given the diagnosis, as he initially thought his symptoms were suggestive of heart attack. He did not expect a diagnosis of a chronic disease, a fact which caused anxieties once his diagnosis was explained to him. Moreover, the fact that the patient had no control over the conversation regarding the diagnosis, which accidentally involved the partner too early, was also highlighted by the patient as a cause of anxiety. In fact, the patient reported being more concerned about his partner's feelings than his own feelings. Furthermore, the fact that the partner went into caring mode soon after the diagnosis is drastic and is an example of the difficulties and anxieties that relatives of heart failure patients may face. In this case, the patient reported no signs of depression. However, the anxiety and adaptation of a new diagnosis of heart failure can often be too difficult to accept and can often impact the patient's behaviour or even lead to depression. A study has recently evaluated the role of anxiety, as well as the role of the cooccurrence of depression and anxiety, in relation to mortality in coronary heart disease (CHD) patients [3]. The results showed an association with mortality for all investigated variables (anxiety alone HR 1.83, depression alone HR 1.66, and both anxiety and depression HR 3.10). Hence, the results of this study suggest that heart disease patients who have* anxiety* have twice the risk of dying from any cause compared to those without anxiety, whereas patients with both anxiety and* depression* have triple the risk of dying. Moreover, the cooccurrence of these psychosocial factors could act as markers of increased mortality risk. A more recent meta-analysis [4] of the association between anxiety and mortality in patients with coronary artery disease (CAD) showed that, in unadjusted analyses, anxiety was associated with a moderate increase in mortality risk (odds ratio 1.21 per SD increase in anxiety). However, when adjusting for covariates, nearly all associations became nonsignificant. In sensitivity analyses, anxiety was associated with an increased risk of poor outcomes in the stable CAD, but this was not the case for patients who had just suffered an acute coronary syndrome. The authors concluded that the analyses confirm that anxiety is associated with increased risk of mortality in patients with CAD; however, this relationship is not as strong as that of depression and may be explained partly by other clinical factors.

Depression is a common comorbid condition in patients with heart failure, with reported prevalence rates from 13% to 77.5%, depending on the definition and assessment strategies [5]. A recent review by Herr et al. [6] examined the relationships between heart failure related symptoms and demonstrated correlation between several heart failure symptom pairs, such as depression and anxiety, depression and fatigue, depression and pain, fatigue and pain, dyspnoea and depression, and dyspnoea and sleep disturbance.

Depression also impacts the partner and family of heart failure patients. Partners caring for an individual with heart failure are often required to assist with a range of activities of personal care (washing, bathing, and aiding mobility), logistics of daily life (shopping, housekeeping), and general health activities like dietary adjustment, clinic visits, ensuring provision of medications, and monitoring symptoms [7]. Moreover, partners may be exposed to clinical crises of their partner which may cause personal anxiety and stress. It has been shown that anxiety and depression of the patient or the partner influence both their own quality of life and that of the other, an observation suggesting dyadic dynamics [8].

Although in this case there was not a formal diagnosis of depression, it is useful to consider the relationship between depression and heart failure. Depression is a strong psychosocial predictor of repeated hospitalizations for heart failure with results from the Heart Failure Adherence and Retention Trial (HART) showing that, compared with nondepressed individuals, those with depression were hospitalized for heart failure 1.45 times more often (*p* = 0.006), even after controlling for physician adherence to evidence-based medications and patient adherence to heart failure drug therapy and salt restrictions [9]. These findings demonstrate the importance of screening for depression early and repeatedly in the course of heart failure management.

Recent findings indicate fewer than 25% of cardiac patients with major depression are diagnosed as depressed, and only about half of cardiac patients diagnosed as depressed receive treatment [5]. Two basic methods are used to assess depression: structured diagnostic interviews conducted by trained clinicians and symptom inventory self-reports by patients [7] in either in- or outpatient setting. Two examples of interviews to detect major depression include the Structured Clinical Interview for DSM-V Axis I Disorders (SCID-I) and the Diagnostic Interview Schedule (DIS). The purpose of symptom inventory self-reports is to provide quantification of the number and severity of depressive symptoms a person is experiencing during a specified time in relation to the continuum of possible symptom experience; the scores indicate the relative strength of the symptoms.

A useful tool to measure depression in patients with heart failure is the Cardiac Depression Scale (CDS). In a study by Ski et al. [10], the CDS was administered in face-to-face interviews to 603 patients with heart failure; the results showed that the CDS has a hierarchy of items which can be interpreted in terms of the increasingly serious effects of depression occurring as a result of heart failure.

The use of validated questionnaires in heart failure by health care practitioners is crucial for the more frequent identification of patients with poor autonomy and poor confidence in self-management and with low quality of life. This can lead to the early identification of patients with coexisting depression that will require additional resources.

Other examples of questionnaires that can be used include the Revised Illness Perception Questionnaire [11], the Self-Care Heart Failure Index [12, 13], the Hospital Anxiety and Depression Scale [14], the Minnesota Living with Heart Failure (MLHF) Questionnaire [15], the European Heart Failure Self-Care Behaviour Scale [13], and the Beliefs about Medicines Questionnaire [16].

It has also been proposed that biological, medical/psychiatric, psychological, and social aspects of morbidity and mortality in heart failure can be integrated in a holistic model [7]. Biological aspects may include age, sex, and ethnicity; social aspects may include social support, pet ownership, and socioeconomic status; psychological aspects can include stress, anxiety, and depression. Several pathological patterns corresponding to the pathogenesis of heart failure have been suggested in depressed patients [7]. These may include neurohormonal activation, for example, activation of noradrenaline, renin-angiotensin-aldosterone, vasopressin, and endothelin 1, as well as hyperreactivity of the hypothalamic-pituitary-adrenal axis, leading to increased release of cortisol into the bloodstream. Other pathological mechanisms may involve hypercoagulability, autonomic neurocardiac dysfunction, and cytokine release. All these factors need to be taken into account in order to effectively manage patients with heart failure. Particular areas that can be addressed may include sleep, as well as psychological elements such as isolation, interest in life matters outside illness, perceptions, and self-image as well as contextualisation of illness. Moreover, family support should be provided when necessary.

Poor adaptation to a new diagnosis of heart failure, along with the anxieties associated with this diagnosis, can often lead to depression. Several approaches exist to treat depression in heart failure; this can include general measures, psychotherapy such as cognitive behavioural therapy (CBT), and pharmacotherapy.

### 4.1. Nonpharmacological Treatment

In terms of nonpharmacological treatments, exercise is widely considered as helpful in relieving depression in patients with heart failure, as well as improving functionality [17]. CBT focuses on how a person's thoughts and feelings affect their behaviour. CBT involves providing rationale for treatment; providing highly structured and clear plans for change, including the provision of a sense of control; providing feedback and support so that the individuals can attribute improvement to their own abilities and efforts; and teaching skills that increase personal effectiveness and independence [5]. Mindfulness should also be considered, as it can allow patients to be more aware of the thoughts and feelings experienced.

### 4.2. Pharmacological Treatment

In terms of pharmacological treatments, trials have focused on SSRIs which have a better side-effect profile compared to tricyclic antidepressants. An example of such a study is the SADHART-CHF (Sertraline Against Depression and Heart Disease in Chronic Heart Failure) trial. This was a was a randomized, double-blind, placebo-controlled trial of sertraline 50 to 200 mg/day versus matching placebo for 12 weeks, which included 469 patients 45 years or older with heart failure and clinical depression; although sertraline was safe in patients with significant heart failure, treatment with sertraline compared with placebo did not provide greater reduction in depression or improve cardiovascular status [18]. Other studies have shown contradicting results; the large multicentre SADHART study (Sertraline Treatment of Major Depression in Patients with Acute MI or Unstable Angina) was a randomized, double-blind, placebo-controlled trial conducted in 40 outpatient cardiology centers and psychiatry clinics in the United States, Europe, Canada, and Australia and involved 369 patients with acute myocardial infarction (MI) or unstable angina and major depressive disorder [19]. After a 2-week single-blind placebo run-in, patients were randomly assigned to receive sertraline in flexible dosages of 50 to 200 mg/d or placebo for 24 weeks. The results demonstrated a relative efficacy and safety of sertraline. These conflicting results suggest that more trials are necessary to determine the role that SSRIs can have in heart failure or to identify patients more appropriately who may or may not benefit from pharmacotherapies for overt depression. Given the neurohormonal disturbances associated with depression in heart failure, it would be interesting to investigate whether certain biomarkers can be used to identify patients that are likely to benefit.

### 4.3. The Role of Sleep

The role of sleep pattern disturbance in heart failure is starting to be investigated. Many patients with heart failure are unable to sleep in the night-time initially due to symptoms of dyspnoea as fluid retention in the legs can move into the lungs, but often even after oedema is controlled, the memory of breathlessness is considered akin to a fear of dying [20]. These patients often prefer to attempt to sleep in the daytime but events do not allow a deep or long enough sleep to counter fatigue and so a vicious cycle starts forming. Sleep disturbance may be related to the pathophysiological processes associated with heart failure and its presence leads to excess morbidity and mortality, decrements in quality of life and functional performance, and negatively affecting self-care capacity and self-care behaviours [21].

### 4.4. Patient's Beliefs about Heart Disease

A further, deeper issue is the appreciation of the diagnosis of heart failure. Denial of illness in the medical setting is a common initial response to a life-threatening illness, which can be either a deliberate or an unconscious process. It has been suggested that denial of illness is adaptive during acute hospital recovery but is maladaptive in the long run after hospital discharge [22]. Our patient demonstrated responses that may be related to denial; rather than focusing on his own feelings, he chose to focus on his partner's reaction, a form of coping and denial. He also decided to expedite his marriage; was this a way of adaptation and acceptance of his diagnosis, or was this just a reaction relating to denial? Moreover, he had perceptions about his heart failure that may also suggest denial; for example, he felt surprised that he was told he had heart failure but yet only experienced arrhythmias but no pain. Denial may increase the health risks of the patient and jeopardise the beneficial effects of treatment, for example, when medical care is refused; hence, rapid evaluation of the most pressing problems, explicit psychodynamic formulation of the dominant conflicts, creation of a practical program of management, and active participation by a psychiatrist can ensure that the patient receives the best possible clinical care [23].

### 4.5. Self-Management of Heart Disease

The appreciation of how the condition can influence treatment adherence and hence clinical outcomes is another concept to be taken into consideration. A recent study by MacInnes [24] indicated three factors were significant predictors of positive self-care: medication knowledge (*β* = 0.319, *p* = 0.003), a belief in the illness having serious consequences (*β* = 0.258, *p* = 0.008), and the impact of medication use on lifestyle (*β* = 0.231, *p* = 0.03). The author suggested that illness representations and treatment beliefs should be routinely explored in patients with heart failure to inform targeted interventions designed to correct misconceptions and enhance self-care. A further factor that may influence decision making in chronic heart failure is memory and cognitive dysfunction, which may be associated with poorer self-care management behaviours, resulting in worsening of heart failure symptoms and hospitalization for acute management [25]. However, heavy workload and time limitations may impose difficulties in addressing these issues (by the doctors and other health professionals). For example, medication knowledge could involve discussion between the pharmacist and the patient. Although the pharmacists would have checked the drug chart and would have seen the patient daily, the patient did not mention any relevant discussions. Moreover, the patient has not mentioned discussing any medication beliefs with nurses administering the medications, nor any discussions with doctors regarding medication knowledge or impact of medication on lifestyle.

## 5. Conclusions

We have used a case study to discuss issues relevant to a new diagnosis of a chronic disease like heart failure. This case demonstrates the subsequent psychological impact on the patient, as well as on carer and family. Depression and anxiety are often found to be comorbid conditions in patients with heart failure. Various tools can be used to screen for depression in patients with heart failure. Both pharmacological and nonpharmacological options are available. Rapid evaluation of ongoing problems and active participation by a psychiatrist can ensure that the patient receives the best possible clinical care. Management should include consideration of the role of sleep, the patient's beliefs about heart disease, and self-management of heart disease.
